# Emerging Stem Cell Therapies: Treatment, Safety, and Biology

**DOI:** 10.1155/2012/521343

**Published:** 2012-08-02

**Authors:** Joel Sng, Thomas Lufkin

**Affiliations:** Stem Cell and Developmental Biology, Genome Institute of Singapore, 60 Biopolis Street, Singapore 138672

## Abstract

Stem cells are the fundamental building blocks of life and contribute to the genesis and development of all higher organisms. The discovery of adult stem cells has led to an ongoing revolution of therapeutic and regenerative medicine and the proposal of novel therapies for previously terminal conditions. Hematopoietic stem cell transplantation was the first example of a successful stem cell therapy and is widely utilized for treating various diseases including adult T-cell leukemia-lymphoma and multiple myeloma. The autologous transplantation of mesenchymal stem cells is increasingly employed to catalyze the repair of mesenchymal tissue and others, including the lung and heart, and utilized in treating various conditions such as stroke, multiple sclerosis, and diabetes. There is also increasing interest in the therapeutic potential of other adult stem cells such as neural, mammary, intestinal, inner ear, and testicular stem cells. The discovery of induced pluripotent stem cells has led to an improved understanding of the underlying epigenetic keys of pluripotency and carcinogenesis. More in-depth studies of these epigenetic differences and the physiological changes that they effect will lead to the design of safer and more targeted therapies.

## 1. Introduction

Mammals are complex organisms populated by a cosmopolitan city of cells. Resembling the individual components of a metropolis, cells are the essential building blocks of all tissues and organs in an organism, ranging from the delicate construction of the inner ear to the sturdy femur. Like bricks in a skyscraper, the identity, role, and position of each cell must be carefully regulated to ensure the development of functional organs. However while buildings have to be designed and constructed, some bricks in each multicellular organism can mediate self-renewal and are commonly identified as stem cells (SCs). Embryonic SCs (ESCs) are pluripotent progenitors that retain the capacity to form cells from the three germ layers. These cells rely on a group of transcription factors that regulate a network of genes required for their maintenance and proliferation. Of these transcription factors, the activity of *Sox2*, *Oct4*, *Nanog*, and *Klf4* is most critical for the maintenance of ESCs [1–3]. *Sox2 *is a member of the SRY-related HMB-box family and maintains ESC pluripotency by inducing *Oct4* expression [4]. Oct4 and Sox2 coexpression then induces the formation of binary complexes that bind to their respective enhancer elements for positive self-regulation [5]. Oct4 also interacts with various Sox transcription factors like Sox2, Sox4, Sox11, and Sox15 for coregulation of genes like *Fgf4, Lefty1, Fbx15, Utf1, *and* Nanog *via *Oct-Sox* enhancers [4, 6–11]. *Nanog *is a homeobox gene which is initially expressed monoallelically in 2–8 cell stage blastomeres and biallelically expressed only in the pluripotent inner cell mass as the embryo matures [12]. Hence monoallelic *Nanog *expression seems to encourage differentiation while biallelic *Nanog* expression maintains pluripotency and is a key regulator in early embryonic development. *Klf4* assists *Oct4* and *Sox2* in regulating various genes including *Lefty1 *expression, maintains stem cell pluripotency, and has been implicated in differentiation and proliferation [11, 13–16]. Further validating the importance of these transcription factors, experiments have shown that the overexpression of Sox2, *Oct4*, and *Klf4* can initiate the reprogramming of adult differentiated cells into *Nanog* expressing induced pluripotent stem cells (iPSCs) [17].

## 2. Induced Pluripotent Stem Cells

Since the pioneering experiments that demonstrated the possibility of inducing iPSCs from mouse fibroblasts via retroviral transduction in 2006, increasing interest in iPSCs has led to the discovery of other alternative methods for producing iPSCs [18]. Transduction via retroviral and lentiviral vectors was among the first methods for generating iPSCs [19–21]. However notable disadvantages of these widely used protocols are that the process results in integration of exogenous genetic material, such as the protooncogenes *c-Myc*, in transformed iPSCs which may increase the risk of tumorigenesis in iPSC-based therapies and the low transformation efficiency of adult cells to iPSCs (0.001–2%) [22]. More recently, transfection of modified mRNA has been shown to reprogram fibroblasts to iPSCs with an efficiency of up to 4.4% without genomic integration of extracellular DNA [23]. More research into miRNA sequences has also led to the identification of the *miR302/367* cluster which, when combined with lentiviral transduction, demonstrated 10% efficiency for reprogramming fibroblasts to iPSCs [24]. These improvements for generating iPSCs could lead to the development of a higher throughput system to obtain stem cells that more closely resemble adult stem cells and ESCs.

The utilization of iPSCs in therapy design and research possesses several distinct advantages over ESCs. Firstly since iPSCs are derived from the patient, there will be a reduced risk of immune rejection compared to allografts or xenografts from ESCs. In addition, it is easier to obtain iPSC precursors from patients compared to the difficulty and ethical concerns associated with the derivation of ESCs. Finally iPSCs are epigenetically different from ESCs and retain predisposition to redifferentiate into their original cell type [25]. This iPSC epigenetic memory could be harnessed to generate cell-specific types that cannot be easily obtained from ESCs.

The reprogramming of iPSCs from normal somatic cells necessitates a complex epigenetic transformation. To appropriately design the perfect iPSC, it is essential to gain a better understanding of epigenetic differences between iPSCs and ESCs. Recent research has provided insight into the epigenetic memory of iPSCs, a signature artifact from parental cells and the reprogramming process which restricts the ability of iPSCs to differentiate and form cells from alternative lineages [25, 26]. These iPSC epigenetic signatures such as differences in CG methylation and histone modification near developmental control genes can be transmitted to their progeny even after differentiation and may affect the function of iPSC-derived cells [27]. Additionally, the role of chromatin-modifying enzymes has also been shown to influence the effectiveness of iPSC reprogramming and is essential for determining cell fate [28, 29]. Hence the residual epigenetic memory of iPSCs has to be completely reset to resemble ESCs before they can be classified as truly pluripotent stem cells. Some molecular and environmental tools which can assist in epigenetic alteration include compounds like sodium butyrate which can modify H3K9 acetylation and CpG demethylation of specific promoters regulating various genes (*Dppa5*, *Ddx43*, *Rcn3*, *Sp5*) [30], DNA methyltransferase inhibitors (e.g., valproic acid, 5-aza-cytidine) which can be designed to inhibit specific methyltransferases for overcoming barriers to DNA methylation reprogramming [31, 32], antioxidant mixtures (e.g., *Withania somnifera* extract), and kinase inhibitors (e.g., tyrosine kinase inhibitors) which can inhibit phosphorylation of various genes [33, 34]. Standardizing the in vitro microenvironment for cultured stem cells is also essential for generating the desired epigenetic expression. Owing to the different culture conditions in various laboratories, even similar stem cell lines can exhibit heterogeneous genetic expression. Greater differences in gene expression exist between various iPSCs and ESCs lines as they can exhibit epigenetic differences even when cultured under similar conditions [35, 36]. Hence close regulation of the in vitro microenvironment will be necessary for proper epigenetic control and maintain stem cell epigenetic homogeneity as even varying oxygen levels alone can induce epigenetic changes that facilitate reprogramming to iPSCs and their directed differentiation [37, 38]. However although a repertoire of epigenetic tools are available, additional research to identify and map the epigenetic ground state of ESCs will be essential for their proper application to reset these inherent iPSC signatures.

Genetic mutations and differences inherent in both iPSCs and other stem cells cultured in in vitro conditions also have to be evaluated before they can be safely utilized in medical therapies. While some studies have indicated that a subset of stem cells such as mesenchymal stem cells are able to maintain genetic stability for up to six months [39, 40], more recent larger-scale studies have demonstrated a predisposition to genetic instability and overexpression of protooncogenes in cultured stem cells due to selective pressures of culture conditions and the consequences of reprogramming to produce iPSCs [41, 42]. Hence more research has to be conducted to determine optimal stem cell culture conditions to prevent undesired genetic abbreviations from occurring. It is also essential to develop reprogramming protocols that generate genetically stable iPSCs. Finally the genetic integrity of all stem cells should be verified before they are utilized in therapies so as to reduce the chance of tumorigenesis in patients.

## 3. Adult Stem Cell Therapies

Despite the limitations in understanding stem cell differentiation and iPSC reprogramming, there has been some progress in verifying the safety of adult stem-cell-based therapies for several diseases. This process is important because many genes activated in stem cells or considered useful in inducing iPSC formation are protooncogenes, and this raises the possibility that stem-cell-based therapies may increase the risk of cancer in patients. For example, the four transcription factors commonly utilized in iPSC reprogramming *Sox2*, *Oct4*, *Nanog*, and *Klf4* have been linked to carcinogenesis, increased cancer malignancy and tumor drug resistance and are overexpressed in many cancers and cancer stem cells [43–51]. Additionally the inactivation of tumor suppressor genes like *p53* exhibits similar effects by encouraging iPSC formation at the expense of increased risk of tumorigenesis and genetic instability [52].

## 4. Mesenchymal Stem Cells

Multiple studies have been conducted in an attempt to verify the safety and effectiveness of various stem cell therapies. Some of these successful studies utilize adult stem cells such as bone-marrow-derived mesenchymal stem cells (MSCs) in trials of various regenerative therapies. These cells were first described as MSCs due to their ability to differentiate and form various mesenchymal cells such as bone and cartilage cells [53]. MSCs form a surprisingly heterogeneous cell population, and the subset of MSCs extracted from bone marrow alone displays a wide variety of cellular morphologies and antigen markers [54]. Human-marrow-derived MSC autografts were one of the first successes in stem cell therapies as there is minimal chance of immune rejection due to their intrinsic immunomodulatory properties [55]. In addition, these MSC transplantations do not typically result in teratoma formation when tested in clinical trials and are relatively safe compared to ESCs and iPSCs which readily form teratomas [56]. In one such study, 41 patients who underwent bone-marrow-derived MSC transplant for joint repair were examined for tumor and infection symptoms for between 5 and 137 months, and no abnormalities were detected [57]. Another study of MSC transplantation for orthopedic therapy involving several hundred patients over a period of 1-2 years also argues that these transplantations are unlikely to increase the risk of carcinogenesis [58]. Other studies have also indicated that MSC transplantation is safe and has led to improved prognosis for orthopedic ailments [59, 60]. The safety of MSCs therapies for myocardial infarction has also been accessed, and patients show improved cardiovascular prognosis [61]. MSCs have also been utilized in organogenesis for lung reconstruction. In one study patient's MSCs and epithelial cells were combined with donated trachea cartilage in a bioreactor to form a functional graft that was then successfully used to rescue patient lung function without immune rejection [62]. More recently, improved tissue engineering methods have reduced the time required for graft generation from 3 months to 3 weeks allowing patients requiring more urgent transplants to be treated [63]. Besides their role in bone and cartilage repair, other MSC-based therapies have also been evaluated for their safety and varying levels of effectiveness for treating various conditions including stroke, multiple sclerosis, diabetes, and kidney transplantation, in other clinical trials [64–68].

MSCs can also be extracted from adipose and synovial tissues, peripheral blood, skeleton muscles, and neonatal tissues like the umbilical cord [69]. Adipose tissue is a rich source of MSCs which have been used to stimulate the regeneration of bones and cartilage tissues in humans and can partially mediate the effects of osteoarthritis and osteonecrosis [70]. The use of adipose MSCs is advantageous as they can be readily extracted via liposuction of adipose tissue which is a minimally invasive procedure and purified via established protocols [71, 72]. Hence adipose MSCs could be considered as a viable source of stem cells if the patient is unable to undergo bone marrow MSC extraction. MSCs can also be isolated from synovial fluid in humans and animals [73]. Initial exploratory studies in rabbits have proven that treatment with synovial MSCs can prevent degeneration of the intervertebral disc [74]. In addition some studies have argued that synovial MSCs may have greater therapeutic effects compared to MSCs derived from other sources due to an increased ability to proliferate, differentiate, attach to damaged tissue, and accelerate healing in animal models [75–77]. Some progress has also been made in engineering in vitro tissue constructs to expedite implantation of synovial MSCs [78].

While MSCs extracted from different regions exhibit similar differentiation potential and therapeutic effect, some distinct traits do exist. Human MSCs derived from marrow express different cellular markers from adipose MSCs. For example, adipose MSCs express higher levels of CD49d, CD34, and CD54 while marrow MSCs express higher levels of CD106 [79]. These naturally occurring differences in homing and mobilization markers could be exploited for more targeted stem cell therapies. The differentiation potential of MSCs also varies. For example, while marrow-derived MSCs have a higher chondrogenic potential, adipose MSCs differentiate to form cardiomyocytes more readily [80, 81]. More in-depth studies of these phenomena could reveal the epigenetic regulators that prime stem cell differentiation and homing and increase the repertoire of tools available to control stem cell specificity, differentiation, and proliferation. Further clinical trials will need to be carried out to verify the therapeutic effectiveness and safety of MSC-based therapies in humans.

## 5. Hematopoietic Stem Cells

Hematopoietic stem cells (HSCs) have also been widely utilized in experimental therapies with pioneering studies of HSC transplantation (HSCT) dating back to the 1950s [82]. HSCs consist of a surprisingly heterogeneous population of multipotent stem cells which collectively possess the potential to form all blood cell types [83]. HSCs can be purified from bone marrow cells by selecting for cells possessing various expression markers like membrane glycoprotein Sca-1 and tyrosine kinase receptor c-Kit (CD117) and lacking terminal differentiation markers [84]. HSCT is most commonly utilized in therapy for various blood- and bone-marrow-related cancers such as leukemia and multiple myeloma. A retrospective analysis of 586 adult T-cell leukemia-lymphoma patients who received allogeneic hematopoietic stem cell transplantation revealed that HSC therapy was effective for improving long-term survival in these patients [85]. Multiple myeloma has also been successfully treated with a combination of HSCT, chemotherapy, and total body irradiation since 1986 [86, 87]. The development of combination HSCT therapies involving newer drugs like lenalidomide, pegylated liposomal doxorubicin, dexamethasone, and bortezomib continues to improve progression-free survival and overall survival rates of these patients [88–91]. However although allogeneic HSCT can improve patient survival and cancer remission through the induction of acute graft-versus-tumor effect (GVT), this process remains poorly understood and may involve various transplanted cell types including T cells, natural killer cells, and B cells [92, 93]. In addition, although full donor chimerism after HSCT reduces the risk of cancer relapse and progression, HSCT is also associated with several negative side effects including graft-versus-host disease (GVHD) which may lead to lethal complications in patients [86, 92]. HSCT patients with chronic GVHD may also be more likely to suffer from secondary tumors such as basal cell skin cancer, oral squamous cell carcinoma, sarcoma, and adenocarcinoma [94, 95].

HSCT has also been tested in other experimental therapies including lysosomal storage diseases and other metabolic diseases such as Hurler syndrome and X-linked adrenoleukodystrophy leading to improved patient survival rates [96–98]. Recently, HSCT involving a CCR5 *δ*32 homozygous donor also resulted in the first successful cure of HIV and has renewed interest in the search of a remedy for this chronic epidemic which has contributed to over 30 million deaths worldwide [99]. In summary, although recent advances in surgical techniques and drug regimens have led to improved HSCT survival rates and disease remission in patients, potential complications related to HSCT have restricted its use to patients with life-threatening diseases. Hence further studies will be essential for enhancing the safety and effectiveness of HSCT. In particular, a better understanding of how the positive effects of GVT can be enhanced and separated from negative side effects like GVHD could lead to major breakthroughs in currently available therapies.

## 6. Neural Stem Cells

The therapeutic potential of neural stem cells (NSCs) has also received considerable interest. Studies have proven that NSCs can be isolated from the mouse bone marrow, striatum, and NSC lines with a stable chromosome number and have also been generated from human iPSCs [100–103]. In addition, NSCs can be reprogrammed into iPSCs by *Oct4* overexpression to generate other cell types [104]. However some studies have proposed that NSCs may be predisposed to culture-induced mutations that could limit their therapeutic utility [105, 106]. Hence more studies should be undertaken to identify suitable in vitro culture conditions for maintaining NSC genetic and epigenetic stability. Due to these limitations, evaluations of the therapeutic effectiveness of NSCs have mainly been performed in animal trials. Several of these studies have documented the effectiveness of NSCs for alleviating the symptoms of multiple sclerosis in experimental autoimmune encephalomyelitis (EAE) mouse models. These studies indicate that NSCs are able to enter the central nervous system, form brain cells, promote neuroprotection, and encourage remyelination [107–110]. A closer look into the role of NSCs in adaptive immune regulation has also revealed that NSCs release a morphogen (bone morphogenetic protein 4) which prevents dendritic cell maturation and reduces the formation of antigen-specific T cells resulting in limited neuroprotection in EAE mouse models [111]. Further experiments involving transplanting NSCs engineered to produce IL-10 have also demonstrated their enhanced potential for enhancing their immune-suppressive effect for mediating the progress of EAE compared to NSC transplantation alone [112].

The study of how endogenous NSCs can be encouraged to mediate repair has also received considerable interest. NSCs exist in specific stem cell niches in adult mammals such as the ependymal layer and can migrate and differentiate to form functional neural cells in rat EAE models [113, 114]. These ependymal-derived NSCs can also differentiate to form supporting cells like astrocytes and oligodendrocytes [115, 116]. The endogenous NSC-based repair mechanism is governed by complex molecular pathways involving morphogens, neurotransmitters, growth factors, transcription factors, cell surface molecules, and nuclear orphan receptors (for review see [117]). Future minimally invasive regenerative therapies may seek to utilize this endogenous source of NCSs by inducing genetic and pharmacologically directed cell migration and differentiation based on an improved understanding of these molecular pathways. NSCs have also been studied for their therapeutic effects in other neural diseases like fetal alcohol spectrum disorder and lysosomal storage diseases [118, 119], and scaffolds containing NSCs have also been shown to encourage recovery from spinal cord injury in Wistar rats [120].

## 7. Other Adult Stem Cells

Many other different categories of naturally occurring stem cells have been identified and investigated for their therapeutic potentials including, mammary stem cells, intestinal stem cells, inner ear stem cells, and testicular stem cells. Mammary stem cell concentration can be enriched by fluorescence-activated cell sorting of mouse mammary glands for cells that are CD31^−^, CD45^−^, Ter119^−^, Sca-1^low^, CD24^+^, and either CD49f^high^ or CD29^high^ [121, 122]. These multipotent mammary stem cells are able to form functional mammary glands when transplanted in mice, and human mammary stem cells have been isolated in an attempt to better understand carcinogenesis and cancer stem cells [121, 123]. Intestinal stem cells can mediate partial restoration of small intestine function after intestinal resection [124]. More studies to elucidate the molecular pathways regulating this regenerative mechanism may lead to the development of novel regenerative gene therapies for improved intestinal function in patients suffering from short bowel syndrome. Hearing loss due to the loss of cochlea hair cells is another ailment that could be treated with stem cell therapy. In the search for a cure, inner ear stem cells have been identified in both the dorsal epithelium of the cochlear canal and the adult utricular sensory epithelium [125, 126]. These ongoing studies point to an ongoing attempt to identify potential progenitors for hearing restoration and the molecular regulators that guide this process. Testicular stem cell transplantation has been successfully used to restore fertility in mice, pig, and goat animal models and assist in the creation of transgenic animals [127–129]. Further advances in this area may result in therapies for preserving the fertility of cancer patients who are infertile due to the side effects of chemotherapy and radiation therapies. In conclusion these studies demonstrate that while ensuring the safety of adult stem cell therapies remains a key concern, the inherent regenerative capacity of the adult stem cell reservoir can be leveraged to augment the effectiveness of existing medical remedies.

## 8. Conclusion

While the field of stem cell medicine is rapidly maturing, many potential stem cell therapies remain theoretical or restricted to successes in animal trials which may not translate directly to safe human stem cell therapies. In addition, a notable limitation of human clinical trials involving stem cell therapies is that they often only involve a small group of patients or that the studies were conducted over a short period of several years which may be insufficient to access the true risks of carcinogenesis. Hence the results may not be accurate when extrapolated to determine the long-term safety and effectiveness of these therapies in a larger population. Therefore stem cell therapies should continue to be utilized only as a last resort when conventional therapies have failed or are unavailable, and the condition and response of patients undergoing these therapies should be assessed at regular intervals to ensure their safety.

It is also important to note that many currently utilized stem cell therapies and clinical trials have limited impact on alleviating disease symptoms. This is because most current therapies only attempt to treat one aspect of the disease. In addition a limited ability to diagnose the varied underlying causes of similar disease symptoms and the lack of tests to identify the complex differences between individual patients have led to the application of a generic therapy to a heterogeneous population of patients. Consequently better tools to determine the unique genetic and epigenetic factors in each patient that lead to each disease symptom are increasingly essential for maximizing the potential of customized stem cell therapies. Moreover combinational therapies capitalizing on the integration of various approaches such as stem cell transplantation, material science, gene therapy, developmental biology, and pharmacology for simultaneously targeting multiple aspects of each disease will progressively be required for developing next-generation therapies.

An improved understanding of the intracellular molecular pathways regulating stem cell differentiation and new methods to manipulate these pathways to maintain genetic stability induce desired epigenetic modifications, and phenotypic changes will also be essential for guaranteeing the safety and potency of future stem cell therapies. To achieve this, the myriad genetic and epigenetic variations in seemingly homogeneous stem cell populations have to be studied in greater detail, and the impact of each variation on differentiation, proliferation, and pluripotency must be quantified.

Finally a complete understanding of the mechanisms of stem cell signaling and intercellular communication, such as stromal cell-derived factor-1/CXC chemokine receptor-4 signaling for guiding hematopoietic stem cell mobilization, has to be elucidated [130]. Elucidating the language behind these molecular communications will enable the design of stem cells which can be guided to specific locations, evade potential immune rejection, and interact with host cells to accelerate tissue restoration. It will also lead to improvements in tissue engineering of more complex organs that require the precise positioning of many different cell types to function normally.

In conclusion it can be seen that although many pioneering discoveries have transformed the way we understand stem cell function, countless studies are still required on the expanding frontier of stem cell research before a complete mastery of stem cell manipulation for maximum therapeutic potential can be achieved.
